# Preoperative diagnosis of a “humanoid” fetus in fetu using multimode ultrasound: a case report

**DOI:** 10.1186/s12887-020-02389-y

**Published:** 2020-10-19

**Authors:** Hualin Yan, Juxian Liu, Yan Luo, Yang Wu, Lanxin Du

**Affiliations:** 1 Department of Medical Ultrasound, West China Hospital, Sichuan University, No.37, Guo Xue Xiang, Chengdu, 610041 China; 2 Department of Pediatric Surgery, West China Hospital, Sichuan University, No.37, Guo Xue Xiang, Chengdu, 610041 China

**Keywords:** Fetus in fetu, Ultrasound, Contrast-enhanced ultrasound, Neonatal surgery, Vessels

## Abstract

**Background:**

Fetus in fetu (FIF) is a rare congenital anomaly. The preoperative diagnosis of FIF and differentiating it from teratoma and other abdominal tumors can be challenging for radiologists. Clarification of the blood supply and the relationship with the surrounding vessels is especially helpful for successful surgery; however, multimode ultrasound (US) performed for FIF has rarely been explored. Here, we first report a “humanoid” FIF case diagnosed by multimode US examinations, with the use of contrast-enhanced ultrasound (CEUS) for clarifying the blood supply features.

**Case presentation:**

A 25-day-old preterm male infant was referred to our hospital for surgery. The US and computed tomography (CT) examinations led to a diagnosis of teratoma at the local hospital. The laboratory workup at our hospital revealed an elevation of total bilirubin, direct bilirubin, indirect bilirubin, alpha-fetoprotein, and neuron-specific enolase levels. A precise diagnosis and differentiation from teratoma, hepatoblastoma, neuroblastoma and other abdominal tumors were needed. In addition, the blood supply and the relationship with the surrounding vessels needed clarification prior to surgery. Multimode US examinations were performed and the features of a “humanoid” FIF as well as the blood supply for the abdominal lesion of the infant were suggested by grayscale US, color Doppler flow imaging (CDFI), and CEUS. Furthermore, CDFI and CEUS revealed an aorta-like structure and umbilical cord-like blood vessels in the “humanoid” FIF, and the CEUS helped with marking the surface of the infant’s abdominal wall. To the best of our knowledge, this is the first case report of CEUS in FIF, and the blood supply was clearly demonstrated in the FIF. The intraoperative findings confirmed our multimode US findings and revealed a “humanoid” FIF. The infant quickly recovered after the operation and had no positive findings at the 2-year follow-up visit.

**Conclusions:**

Multimode US was helpful in diagnosing the rare FIF without radiation exposure. Specifically, CEUS clearly demonstrated the limb branch vessel-like structures, the abdominal aorta-like structure and the blood supply, which was useful for the FIF diagnosis and for avoiding damage to important vessels during the operation.

## Background

Fetus in fetu (FIF) refers to a malformed, vestigial fetus inside the body of another normally formed fetus, representing a monozygotic, monochorionic diamniotic twin of the autosite. The incidence of FIF is estimated at 0.2 per million births [1]. The FIF can be found in the abdomen, chest, scrotum or other locations, and a “humanoid” FIF is rarer [2]. Some literature has reported FIF diagnosed by grayscale ultrasound (US), contrast-enhanced computed tomography (CECT) and magnetic resonance imaging (MRI) [3–5]. However, there is radiation exposure with computed tomography (CT) examinations, especially for young children, and misdiagnoses have been frequently reported. Moreover, multimode US technologies performed for a FIF diagnosis have rarely been explored. Here, we report in detail a rare “humanoid” FIF case diagnosed with radiation-free multimode US technologies, where we first applied contrast-enhanced ultrasound (CEUS) to clarify the blood supply for the lesion.

## Case presentation

A 25-day-old preterm male infant (34 weeks of gestational age) was referred to our hospital with a one-month history of an abdominal mass. Antenatal ultrasonography revealed an abdominal mass in the fetus one month prior. After birth, the infant experienced abdominal distension and underwent abdominal US examinations. The abdominal lesion was considered as a teratoma at a local hospital.

After admission to our hospital, the physical examination revealed a 5 cm × 4 cm palpable abdominal mass with no concomitant symptoms. The results of the laboratory workup were normal, except the total bilirubin (TBIL) level was 98.7 μmol/L (reference range, 5.0–28.0 μmol/L), the direct bilirubin (DBIL) level was 17.9 μmol/L (reference range, < 8.8 μmol/L), the indirect bilirubin (IBIL) level was 80.0 μmol/L (reference range, < 20 μmol/L), the alpha-fetoprotein (AFP) level was> 1210 ng/ml (the 95% interval of normal serum AFP for 22–28 days premature infant is 1164–118,850 ng/ml [6]), and the neuron-specific enolase (NSE) level was 21.48 ng/ml (reference range, < 15 ng/ml). Echocardiography revealed a patent foramen ovale. Chest radiography revealed no abnormalities.

To obtain the precise diagnosis, differentiate the lesion from teratoma and other abdominal tumors, and clarify the blood supply and the relationship with the surrounding vessels before surgery, detailed, specialized imaging examinations were needed. The infant underwent abdominal multimode US examinations, including grayscale high-frequency US, color Doppler flow imaging (CDFI) and CEUS technology, which used 0.03 ml/kg SonoVue (Bracco, Milan, Italy) with intravenous bolus injection.

**Fig. 1:**
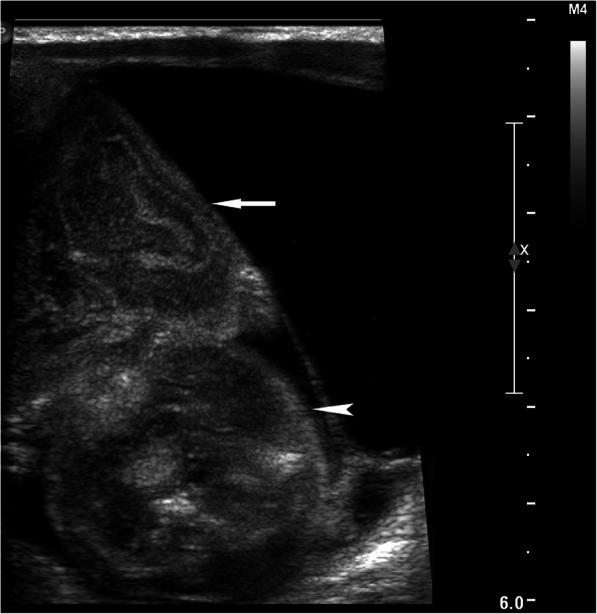
Transverse grayscale high-frequency ultrasound imaging: Ultrasound revealed a 5.5 cm × 5.0 cm heterogeneous, complex cystic and solid intraabdominal mass, which appears as a head (arrow) and body (arrowhead) in shape

**Fig. 2:**
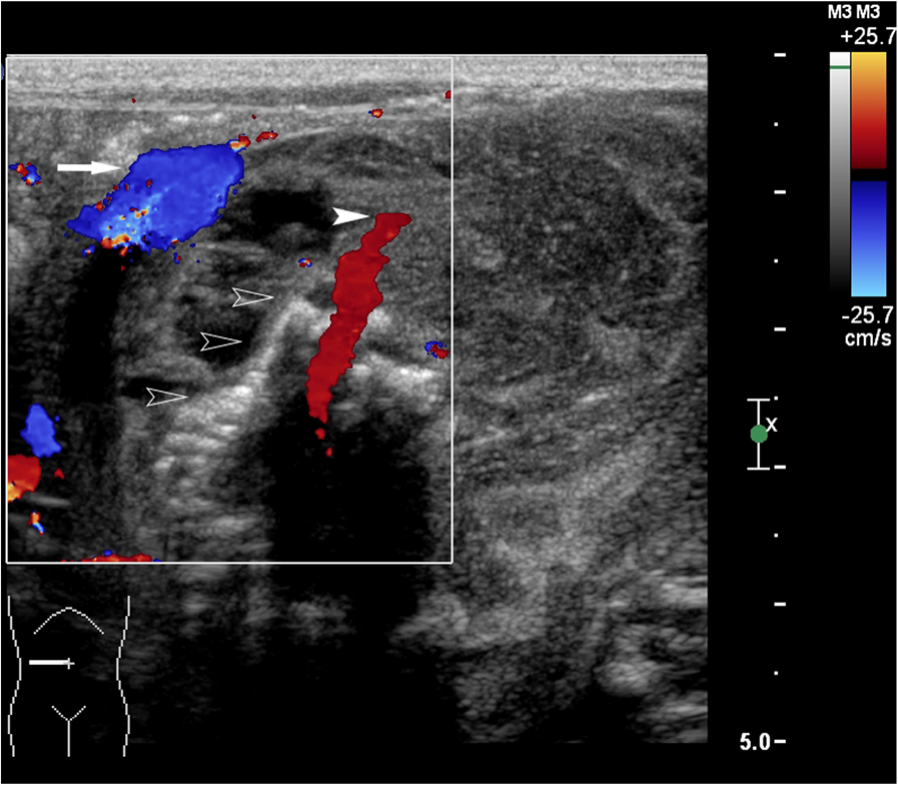
Transverse color Doppler ultrasound imaging: Ultrasound showing an abdominal aorta-like structure (white arrowhead) and an umbilical vessel-like structure (white arrow) within the abdominal mass. Multiple separated, small hyperechoic spots can also be seen, highly organized in a vertebral column-like structure (hollow arrowhead)

**Fig. 3:**
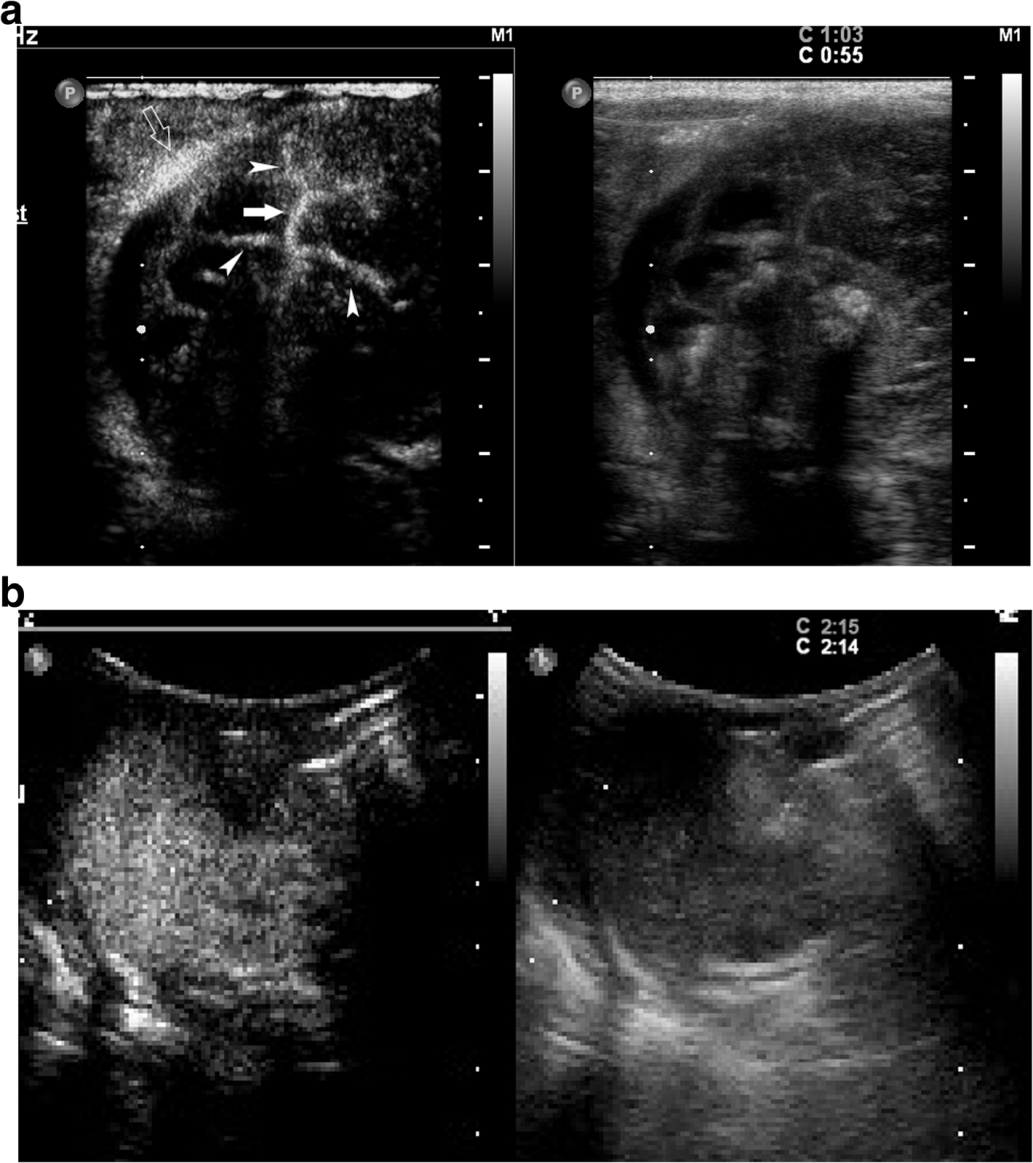
Contrast-enhanced ultrasound (CEUS) imaging (left) and corresponding grayscale ultrasound imaging (right): CEUS clearly showing that the abdominal aorta-like structure (white arrow), limb branch vessel-like structure (white arrowhead), and umbilical vessel-like structure (hollow arrow) in the mass are enhanced at 55 s (**a**), and the mass is persistently enhanced until 2 min and 14 s (**b**)

**Fig. 4:**
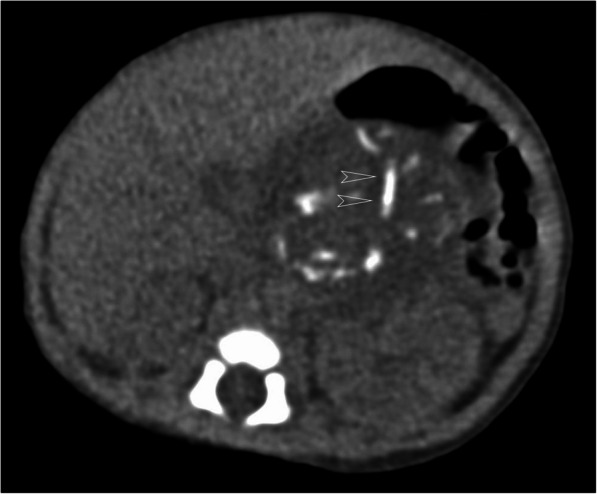
Axial unenhanced CT image of the mass: CT showed a hyperattenuating axial skeleton (arrowhead)

The initial grayscale US revealed a 5.5 cm × 5.0 cm mass in the right upper abdomen of the infant, with a heterogeneous, complex cystic and solid texture. The shape of the solid component of the mass appeared as a fetus with a head and body parts and with multiple separated, small hyperechoic spots highly organized as a vertebral, column-like structure. The lesion had a clear border and regular shape and was surrounded by a well-defined membranous capsule (Fig. 1). CDFI revealed an abdominal aorta-like vessel in the fetal-like solid component of the lesion, which had an arterial spectrum with a high peak systolic velocity of 58.4 cm/s (Fig. 2). Furthermore, an umbilical vessel-like structure was observed on CDFI of the lesion, which was close to the right upper abdominal wall of the infant (Figs. 1 and 2). CEUS clearly showed and further confirmed the abdominal aorta-like structure, with approximately 2 mm in diameter limb branch vessel-like structures and an umbilical vessel-like structure that was approximately 3 mm in diameter, which was the largest feeding vessel of the mass and was connected to the abdominal aorta of the patient (Figs. 2 and 3). A plain CT scan detected a 5.6 cm × 4.6 cm heterogeneous retroperitoneal mass with a hyperattenuating axial skeleton, which corresponded with the hyperechoic vertebral column-like structure on US (Fig. 4). No other positive findings were observed for the liver, gall bladder, pancreas, spleen or kidney on US or CT. Moreover, to avoid a potential bleeding risk by damaging the umbilical vessel-like structure during laparotomy, the structure was positioned and clearly marked on the neonatal surface skin of the abdominal wall under US guidance before surgery.

**Fig. 5:**
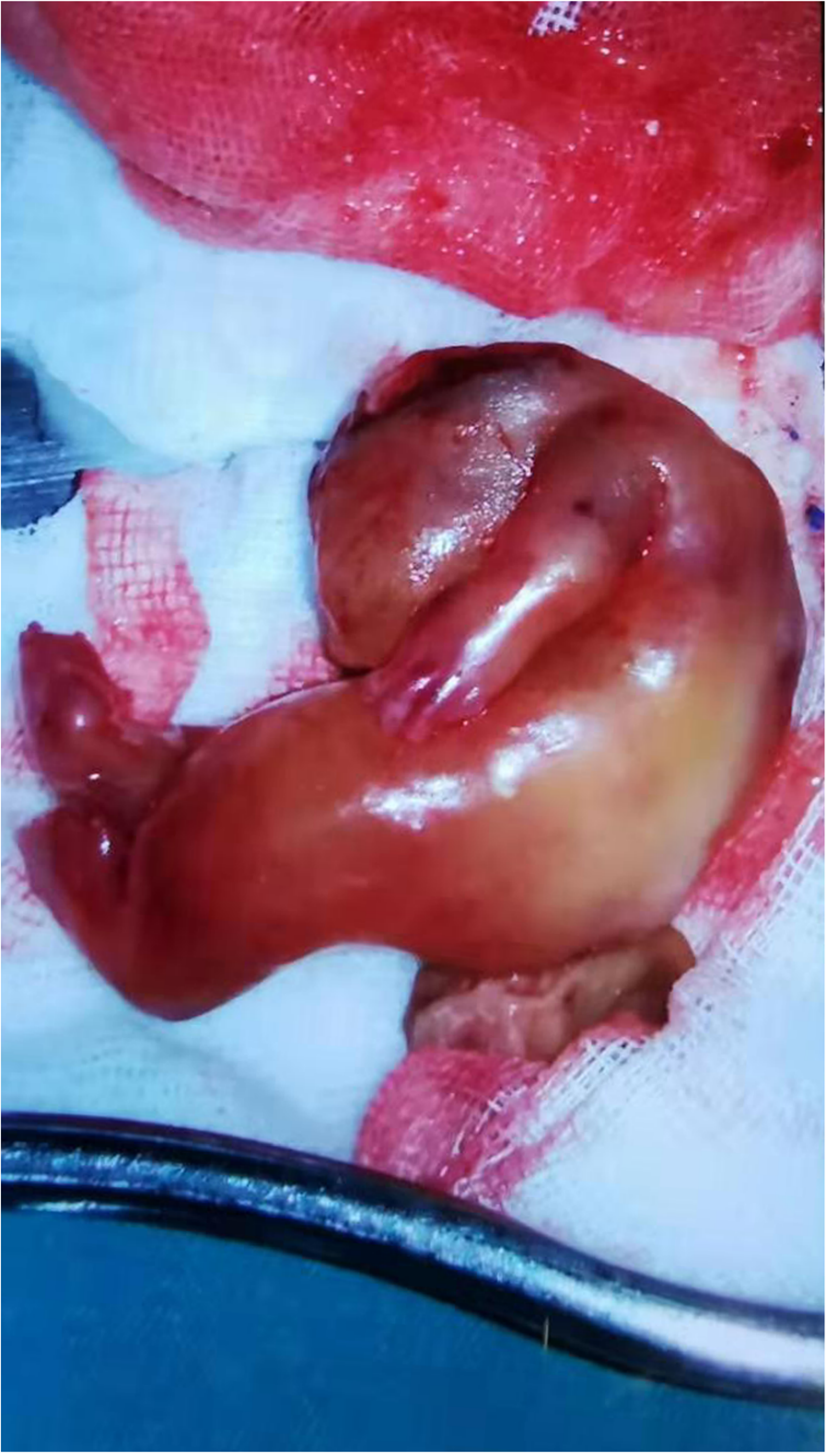
Gross specimen of the mass: The FIF presents a “humanoid” appearance

Given the US findings, an abdominal “humanoid” FIF was diagnosed. The patient underwent laparotomy for mass resection. During the operation, a well-encapsulated, complex cystic and solid retroperitoneal mass approximately 6 cm in diameter was found. A “humanoid” FIF was found in the mass, surrounded by clear, yellow, cystic fluid (Fig. 5). The umbilical vessel-like structure of the FIF was connected to the superior mesenteric artery of the infant, supplying blood to the FIF. The mass was removed en bloc, and the superior mesenteric artery of the patient was repaired. The intraoperative findings supported the diagnosis of FIF, and postoperative histology confirmed it. The premature patient successfully recovered soon after the surgery without any postoperative complications. At the two-year follow-up visit, US showed no recurrence, and the laboratory workup showed normal serum levels of beta-human chorionic gonadotropin (β-HCG) and AFP.

## Discussion and conclusion

Anatomically, FIF has the following characteristics: enclosed within a distinct amniotic sac, partially or completely covered by normal skin, grossly recognizable anatomic parts, and attached to the autosite by a pedicle containing a few relatively large blood vessels [7]. An axial vertebral column and limb buds are seen in most FIFs; other structures, such as dermal, gastrointestinal, and portions of the central nervous system, are commonly found in FIF. Notably, most FIFs are anencephalic and acardiac. The superior mesenteric artery of the autosite is often reported as the feeding vessel to the FIF [2, 4].

According to the reported literature, FIF has the following radiological features. On grayscale US, FIF shows a complex, amorphous mixed echogenic mass with ill-defined solid and cystic components. Hyperechoic vertebral column-like structures can often be detected, similar to our case [2, 3, 8]. CDFI can reveal the largest feeding vessel and rudimentary umbilical stalk of the FIF, as in our case [9, 10]. Importantly, we first reported the contrast enhancement pattern of FIF on CEUS. CEUS clearly demonstrated the main trajectory of the arteries in the FIF, suggesting that the limb branch vessel-like structures originated from the abdominal aorta-like structure and the blood supply was from the autosite. These findings helped to clarify the blood supply of the FIF and mark the umbilical vessels preoperatively, avoiding damage to the vessels and accidental bleeding during the operation. CT scans show a large, well-defined, rounded retroperitoneal mass with solid, cystic and fatty components and can be used to identify the hyperattenuating spinal column and limb buds with long bones, as seen in our case [3, 11]. Prescher L.M. et al. reported that ﻿3D reconstructions and computed tomography angiography (CTA) help in exact anatomical localization and feeding vessel identification preoperatively [2]. On MRI, FIF shows a well-defined mass of mixed high, intermediate and low intensities. Limb buds and spinal axis are usually seen. A cord-like pedicle connected the outer cyst can be seen on MRI [3]. In additional, fetal MRI provided additional information to aid in the prenatal diagnosis of FIF [10].

The laboratory workup in our hospital revealed the elevation of TBIL, DBIL, IBIL, and NSE levels. Therefore, several differential diagnoses should be considered, including teratoma. A teratoma comprises several different types of tissue, such as hair, muscle, teeth, and bone. Its prognosis varies drastically [12]. On US, it often manifests as a cystic, solid or complex cystic and solid mass with hyperechoic calcifications and bone-like structures. CT shows a well-defined mass with cystic, solid and fatty components and hyperattenuating disorganized bony elements [3]. The features that distinguish a FIF from a teratoma are as follows: there must be a separate spinal column and symmetrical development around this axis. The organs must have developed in a synchronized manner [13]. In our case, US demonstrated an axial vertebral column-like structure. CDFI and CEUS revealed an abdominal aorta-like structure, limb branch vessel-like structures and an umbilical vessel-like structure. These imaging findings indicate highly developed organs around the axial skeleton, which distinguishes FIF from teratoma. However, there are case reports of a FIF and a teratoma occurring in the same patient and a FIF with a malignant recurrence [14, 15], which supports the argument that there is a pathological overlap of these two entities and they are at different stages of maturation [12].

Other retroperitoneal infant malignant tumors, such as hepatoblastoma, neuroblastoma or Wilms’ tumor, can generally be ruled out by normal findings for the abdominal organs.

In summary, we described a case of a preterm patient with an abdominal mass due to FIF, presenting as a heterogeneous, complex cystic and solid mass in the retroperitoneal area with an axial skeleton, an abdominal aorta-like structure, limb branch vessel-like structures and umbilical cord-like blood vessels on CEUS. This is the first report of CEUS in FIF. Identification of the vascular structures of the mass not only helped in the diagnosis but also guided choosing the appropriate surgical procedure, which resulted in the early recovery of our premature patient. Our case shows that multimodal US (grayscale high-frequency US, CDFI and CEUS) is safe, accessible, and useful in the diagnosis of infant FIF, avoiding radiation exposure. Nonetheless, more experience is needed with the application of multimodal US for diagnosing FIF.

## Data Availability

All data supporting the findings of this article are included with the article.

## Abbreviations

AFP: Alpha-fetoprotein
CEUS: Contrast-enhanced ultrasound
CDFI: Color Doppler flow imaging
CT: Computed tomography
FIF: Fetus in fetu
US: Ultrasound
